# A Repair Technique for Internal Nasal Valve Collapse Using Lateral Nasal Suspension Sutures

**DOI:** 10.3390/bioengineering11111138

**Published:** 2024-11-12

**Authors:** Morgan Davis Mills, Víctor de Cos, Archana Podury, Deborah Watson

**Affiliations:** 1 University of California, San Diego School of Medicine, 9500 Gilman Drive, La Jolla, CA 92093, USA; modavis@health.ucsd.edu (M.D.M.); vdecos@health.ucsd.edu (V.d.C.); apodury@health.ucsd.edu (A.P.); 2 Department of Otolaryngology Head and Neck Surgery, University of California, San Diego, 9300 Campus Point Drive, La Jolla, CA 92037, USA

**Keywords:** internal nasal valve, nasal valve collapse, nasal obstruction, lateral nasal suspension sutures

## Abstract

One of the most common reasons a patient would see an otolaryngologist is for nasal obstruction. This article provides an overview of the physical principles related to nasal airflow as well as the critical role that the internal nasal valve plays in regulating airflow resistance. Common treatment options for internal nasal valve obstruction are discussed as well as an in-depth tutorial on an alternate lateral nasal suspension suture technique for internal nasal valve collapse.

## 1. Introduction

Nasal airflow is regulated by the internal nasal valve (INV), a narrow anatomic landmark at which the majority of airflow resistance occurs [1]. This structure is bordered by the nasal septum, caudal margin of the upper lateral cartilage, nasal floor, and the anterior aspect of the inferior turbinate [2]. The INV marks the narrowest portion of the human airway and typically measures 10 to 15 degrees; however, because of the dynamic feature of the valve, individuals can experience dynamic INV collapse, static INV stenosis, or a combination of both.

The importance of the INV can be understood through two fluid dynamics principles, Poiseuille’s Law and the Bernoulli effect. First, Poiseuille’s law, Q = πΔPr^4^/8 μL, describes the flow of an incompressible fluid through a cylindrical pipe. This equation states that the volumetric flow rate of a fluid (Q) is directly proportional to the radius to the 4th power (r^4^) and pressure difference (ΔP) between the two ends of the pipe and inversely proportional to the viscosity (μ) and length (L) of the pipe. Although not perfectly cylindrical, the nasal airway acts as an irregular pipe through which the principles of Poiseuille’s law can be applied to understand airflow dynamics. This law highlights how small changes in the radius of the INV according to the r^4^ within the formula’s numerator have a significant effect on increasing the cross-sectional area of the INV and reducing airflow resistance. The Bernoulli equation, P + 1/2ρv^2^ +ρgh = constant, describes how the pressure (P) within a moving fluid varies with changes in velocity squared (v^2^), fluid density (ρ), acceleration due to gravity (g), and height (h). When applied to airflow, Bernoulli forces explain the function of the INV during dynamic collapse. On inspiration, as the airflow increases through the nasal valve, it causes a decrease in pressure, which ultimately acts as a vacuum. Without adequate rigidity of the lateral nasal sidewall, the upper lateral cartilages collapse inwardly and contribute to dynamic INV obstruction.

Nasal obstruction due to INV collapse affects approximately thirteen percent of the American population, and its ensuing impairment of nasal breathing results in significant disruption in quality of life by affecting respiratory function during waking and sleeping hours [3]. The INV can be intrinsically vulnerable due to a narrow nose or a deviated septum. A broad spectrum of etiologies including iatrogenic causes, congenital abnormalities, facial nerve weakness, traumatic injuries, and normal aging processes can further compromise the integrity of the upper lateral cartilages and contribute to worsening dynamic INV collapse [4].

There are a variety of methods used to treat INV collapse. One commonly practiced nonsurgical option is the use of Breathe Right Nasal Strips (GlaxoSmithKline, Brentford, London, UK). Breathe Right Strips consist of a flexible, adhesive material with embedded, spring-like bands made with a combination of microcrystalline cellulose and maltodextrin. When applied across the external surface of the middle nasal vault, their adhesive side adheres to the nasal skin, exerting a lateral lifting force on the nasal sidewalls which physically achieves greater airway patency within the nasal cavity. Unfortunately, Breathe Right Strips are only a temporary solution while they are being worn by individuals. They are not able to alter the nasal valve anatomy with adjustments to the stiffness or memory of the INV cartilages with prolonged or chronic usage. Additionally, some studies have suggested that the benefit of these temporary nasal strips may be due to a placebo effect rather than actual improvement in nasal airflow and sleep quality [5].

**Figure 1:**
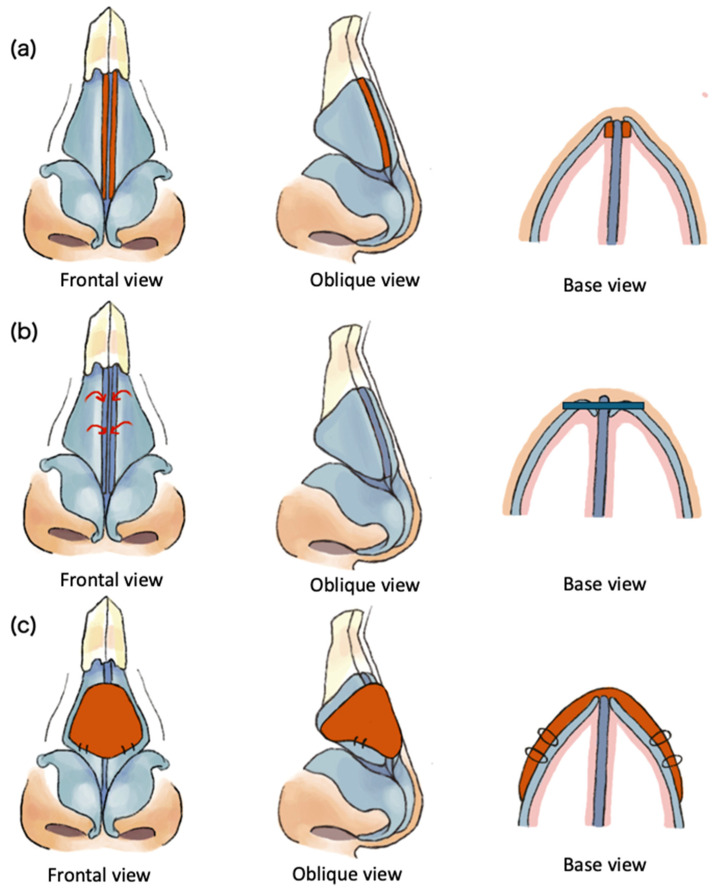
Cartilage grafting techniques for internal nasal valve collapse. (**a**) illustrates the effect of spreader grafts (frontal, oblique, and base views) increasing the width of the angle of the internal nasal valve. (**b**) demonstrates the autospreader graft technique (frontal, oblique, and base views). The medial border of the upper lateral cartilage is detached from the septum, allowing for the upper lateral cartilage to be folded inward and sutured in place to widen the valve. (**c**) illustrates the butterfly graft which spans across both apexes of the internal nasal valve to provide support. The red arrows indicate the direction that the cartilage is folded inwards. The black circles represent sutures through the butterfly graft and upper lateral cartilage.

Alternatively, many surgical approaches targeted at increasing the narrow angle in the INV have been described in the literature and involve cartilage grafting, synthetic implants, and suture techniques. Cartilage grafting or cartilage reshaping techniques include spreader grafts, autospreader grafts, butterfly grafts, alar batten grafts, and alar composite grafts which act to strengthen the lateral side wall or increase the angle of the valve (Figure 1). Synthetic implants include a titanium butterfly implant (Breathe-Implant, Heinz Kurz GmbH, Dusslingen, Germany) and a poly-L-lactide (PLLA) bioabsorbable polymer implant (Latera^®^ absorbable nasal implant system, Stryker ENT, Plymouth, MN, USA). Finally, suspension techniques have the ability to reposition the upper lateral cartilages and have been described by various sources. A transconjunctival approach has been reported, in addition to a Mitek bone anchor technique, flaring sutures, a lateral pull-up technique, and a piriform rim suspension [3,6]. An in-depth literature review by Sinkler et al. [3] concluded that none of these techniques were necessarily superior to one another; therefore, when selecting an approach, physicians must consider a variety of factors that pertain to patient demographics, the current functionality of the patient’s INV, aesthetic results, technical difficulty, prior surgical history, and patient preferences.

In this article, the authors present a how-to approach for an alternate lateral nasal suspension suture technique which could be added to a surgeon’s armamentarium for nasal airway procedures. As with all variations of nasal surgical techniques, careful patient selection can improve a successful outcome.

## 2. Indications for Surgery

All patients were evaluated in the clinic and found to have symptomatic nasal obstruction with a positive modified Cottle maneuver. The modified Cottle maneuver is performed by the direct placement of a cotton-tip applicator at the caudal edge of the upper lateral cartilage, with a slight superolateral displacement of this region, and is considered clinically positive if this intervention results in significant improvement in the patient’s nasal breathing. Surgical intervention will result in the slight widening of the middle vault nasal area; therefore, in addition to standard photos for nasal analysis, intentional discussion with the patient is necessary to demonstrate the effect of the cotton-tip applicator on the nasal valve and to ensure that the subtle change in the overall width of the nose is acceptable to the patient. Medical management is trialed initially, and other causes of nasal airway obstruction, such as allergic rhinitis, are appropriately addressed prior to surgery. As part of the informed consent process, patients are counselled on alternate interventions to address INV collapse including nonsurgical treatments, synthetic implants, as well as a standard septorhinoplasty procedure using cartilage grafts.

## 3. Surgical Technique

### 3.1. Skin Marking and Injection

**Figure 2:**
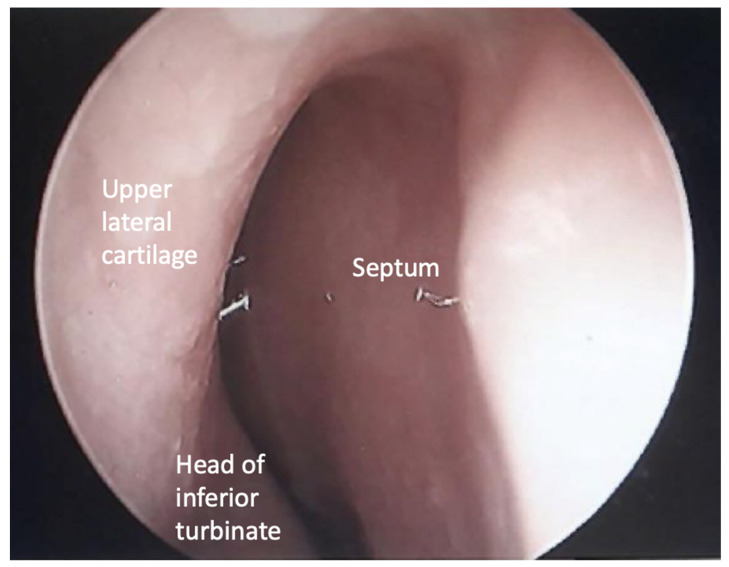
Endonasal view of a narrow internal nasal valve using a zero-degree nasal endoscope.

Patients can be placed under general anesthesia or under monitored anesthesia care in a supine position with IV sedation or local anesthesia alone. No Oxymetazoline or other nasal decongestants are utilized in order to visualize the true baseline of the patient’s INV and nasal mucosa. Once anesthesia is induced, the patient is prepped and draped for the procedure to maximize the sterile technique. A zero-degree nasal endoscope can be used for photo documentation of patency of the patient’s natural INV angle (Figure 2). At this time, a folded cotton pledget can be gently placed posterior to the INV region on both sides of the nasal cavity to reduce the likelihood of scant amounts of blood from dripping into the nasopharynx.

**Figure 3:**
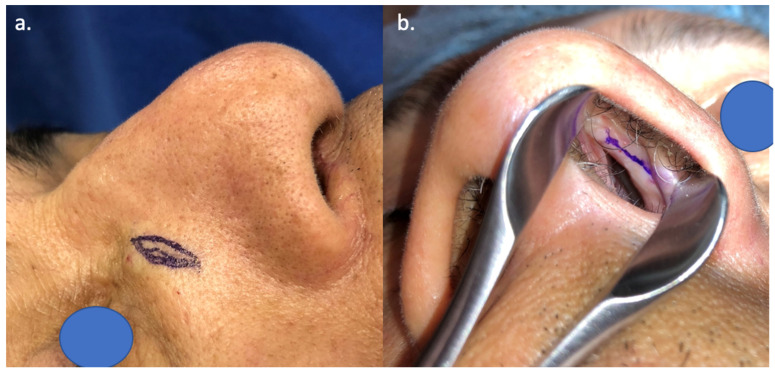
Intraoperative view of (**a**) external fusiform skin excision placement over the nasal bones, parallel to the nasojugal groove, and (**b**) endonasal marking for the superficial scored incision.

Using a nasal speculum to directly visualize the caudal edge of the upper lateral cartilage, the surgeon places a Cottle elevator or Freer elevator against this region to determine the maximal excursion of the upper lateral cartilage. Once the location of maximum INV lift has been identified, the surgeon places a set of fine dotted lines at the caudal edge of the upper lateral cartilage, indicating the location of the endonasal scored incision. With the caudal edge of the upper lateral cartilage still in a lifted position, the surgeon locates the ideal position for an external nasal fusiform skin excision overlying the nasal bones, adjacent and parallel to the nasojugal groove (Figure 3). Fusiform skin excisions range from 10 to 12 mm in length and 3 to 5 mm in width. The skin is then injected with less than 0.5 cc of 1% lidocaine with 1:100,000 epinephrine for local anesthesia and hemostasis, minimizing distortion to the soft tissues.

### 3.2. Skin Excision and Suture Placement

A 15 blade is used to excise the pre-marked fusiform skin excision and to place the superficial scoring incision along the intranasal dotted line at the caudal edge of the upper lateral cartilage. It is recommended to exercise caution and avoid making the endonasal incision too deep in order to prevent the suture from “cheese-wiring” into the soft tissue of the nasal sidewall during the next step. The external fusiform skin is then excised full thickness, with limited undermining of the surrounding skin in the supraperiosteal plane.

**Figure 4:**
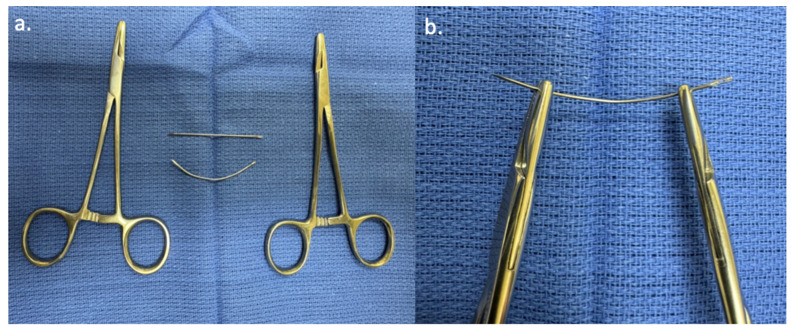
Manual bending of the Keith needle. (**a**) illustrates the initial straight 2-inch Keith needle and the curved needle after manual bending using 2 empty needle drives. (**b**) demonstrates how the needle is bent using the 2 needle drivers.

Next, a 2-inch straight Keith needle (Aspen Surgical Products, Inc., Caledonia, MI, USA) is manually bent in a stepwise fashion by the surgeon, using two empty needle drivers to create a soft curve (Figure 4); this will assist in safely passing the suture into the nasal sidewall and staying clear of the eye. Once a gentle 30- to 45-degree curvature is created in the Keith needle, the free end of a 4-0 Prolene suture on an RB1 needle (Ethicon, Inc., Raritan, NJ, USA) is threaded through the hole at the end of the curved Keith needle. Starting medially in the nasojugal groove incision, the first pass using the Keith needle enters the soft tissue under the edge of the undermined skin and traverses a short distance within the lateral nasal sidewall tissue. It then emerges out of the medial aspect of the score mark made previously in the nasal mucosa. The second pass of the curved Keith needle re-enters through the intranasal score mark approximately 3–5 mm lateral to the earlier exit site. It traverses a short distance again within the lateral nasal sidewall soft tissue to exit the nasojugal groove incision.

**Figure 5:**
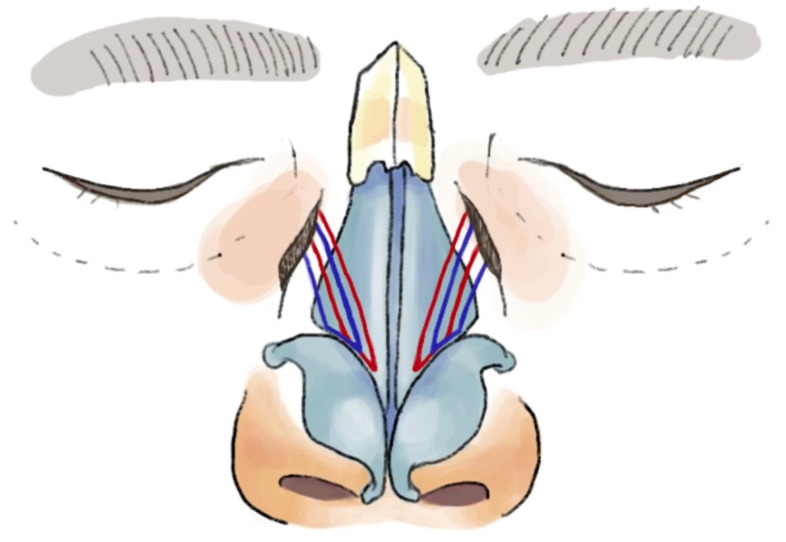
Schematic illustration of lateral nasal suspension sutures. The red line represents the first suture medially placed and the blue line represents the second separate suture. Note the overlapping of the two sutures to reinforce the lifting effect of the lateral side wall.

At this point, the Keith needle is removed and the swaged needle portion of the Prolene suture is passed through the periosteum of the nasal bone to anchor the suture. These steps are repeated with a separate 4-0 Prolene suture to allow the second suture to be placed in a more lateral position to the first one. This permits the two sets of sutures to overlap one another and collectively reinforce the lifting effect of the lateral nasal sidewall (Figure 5).

After the Keith needle is removed, the secondary suture is anchored to the periosteum of the nasal bone using the swaged end of the needle. Now, both sutures are individually and carefully tied with appropriate tension. This maneuver allows for the lifting of the nasal sidewall soft tissue and immediate improvement in the INV. It is recommended that the cut ends of the two Prolene sutures are tucked beneath the undermined skin flap so as to avoid palpable protruding sutures under the skin.

The fusiform excision is closed with interrupted 5-0 Vicryl (Ethicon, Inc., Raritan, NJ, USA) as deep dermal sutures to remove tension from the skin edges. The cutaneous portion can be closed with either a 5-0 Chromic (Ethicon, Inc., Raritan, NJ, USA) or a 6-0 Prolene suture. Depending on the etiology of INV collapse, this procedure can be performed either bilaterally or unilaterally.

### 3.3. Postoperative Care

Minimal postoperative care is required after this procedure. Ophthalmic antibiotic ointment is applied to the external skin incisions for the first 7 days followed by a non-antibiotic petroleum-based ointment or gel until the sutures resorb or are removed. Oral antibiotics are not routinely prescribed, although this can be offered at the surgeon’s discretion. In addition, narcotics are not routinely prescribed, and pain management is well controlled with over-the-counter analgesics. It is also imperative to counsel patients regarding the effect of postoperative edema. Generally, patients will notice an immediate improvement in their nasal breathing before the edema sets in; however, with some patients, this may gradually worsen over the first 48–72 h. For those individuals, it may take a few weeks for improvement.

## 4. Discussion

The concept of nasal valve suspension is based on the physical principle that the negative pressure created by Bernoulli forces during inspiration results in INV collapse due to the medialization of the upper lateral cartilages. Suspension sutures counteract these forces by lateralizing the upper lateral cartilage and increasing the angle of the INV, providing a lateral vector of pull on the nasal side wall, and further stabilizing the overlying soft tissue.

The first documented nasal valve suspension technique was described by Paniello et al. [7] in 1996, whereby the upper lateral cartilages were suspended by the placement of a permanent suture that was retracted laterally and fixated to the inferior orbital rim. Friedman et al. [8] later described a modified technique that used a soft tissue bone anchor system (Mitek anchor) to suspend the nasal valve to the orbital rim. Both methods have proven to be effective in improving nasal valve obstruction, with a 91.7% subjective improvement at 6 months reported in the Friedman study.

The technique described in this paper is rapid, simpler, and equally effective as those in the current literature. Without the need for a bone-anchored implant, patients palpate minimal irregularities beneath the skin. In addition, the technique described in this paper is associated with less than 5% incidence of postoperative skin infections (per senior author) compared to 22% of cases reported with other approaches [9]. One significant modification made with this technique is that the placement of the skin excision tends to be more medially-based, overlying the nasal bone and frontal process of the maxilla instead of more laterally at the infraorbital rim. The authors believe that this placement allows for a more successful superolateral vector of pull that improves the lifting effect of the INV. In addition, with the resultant scar being placed within or parallel to the nasojugal groove, scar camouflage is enhanced, particularly if the patient wears eyeglasses.

There are several advantages to this lateral nasal suspension technique. Patients are still able to achieve improved nasal breathing due to the lateralization of the nasal sidewall even if there is a preexisting septal deviation or inferior turbinate hypertrophy. In addition, this procedure is fast, and can be completed within 30 to 45 min with minimal bleeding risk. This technique may also be a preferred option for more senior patients, those with a history of prior nasal surgeries, patients with thick nasal skin, those who have palpable scar tissue within the nasal skin-soft-tissue-envelope, or patients with nasal valve collapse from loss of facial tone (e.g., facial paralysis).

Complications associated with this technique are rare. Although a permanent suture is used for suspension, there is a possibility of suture failure or breakage from nasal trauma. The lifting tension of the sutures may decrease with the excessive manipulation of the nose by the patient in the post operative period. There is also the risk that the efficacy may gradually diminish over time due to the steady increases in laxity in the nasal framework and surrounding tissues that occur with the aging process. Other minor complications include infection, foreign body reaction to the suture material, and the need for revision surgery. To our knowledge, there have been no major complications in our experience or identified among other reports in the existing literature [8,9].

## 5. Conclusions

This article provides a comprehensive overview of the INV, a review of Bernoulli’s and Poiseuille’s physics principles as it relates to airflow in the nasal cavity, and how the INV has a critical role in nasal airflow regulation. An alternate technique is offered for managing the collapse of the INV with a simple and time-efficient lateral nasal suspension procedure. As a solution for INV collapse, it has comparable functional outcomes to other approaches found in the literature, it eliminates the need for significant surgical dissection, and aligns with aesthetic outcomes for an appropriate surgical patient.

## Data Availability

No new data were created or analyzed in this study.
